# The Effect of Bedding System Selected by Manual Muscle Testing on Sleep-Related Cardiovascular Functions

**DOI:** 10.1155/2013/937986

**Published:** 2013-11-25

**Authors:** Terry B. J. Kuo, Jia-Yi Li, Chun-Ting Lai, Yu-Chun Huang, Ya-Chuan Hsu, Cheryl C. H. Yang

**Affiliations:** ^1^Institute of Brain Science, National Yang-Ming University, No. 155, Section 2, Li-Nong Street, Taipei 11221, Taiwan; ^2^Sleep Research Center, National Yang-Ming University, No. 155, Section 2, Li-Nong Street, Taipei 11221, Taiwan; ^3^Department of Education and Research, Taipei City Hospital, No. 145, Zhengzhou Rd., Datong Dist., Taipei 103, Taiwan

## Abstract

*Background*. Different types of mattresses affect sleep quality and waking muscle power. Whether manual muscle testing (MMT) predicts the cardiovascular effects of the bedding system was explored using ten healthy young men. *Methods*. For each participant, two bedding systems, one inducing the strongest limb muscle force (strong bedding system) and the other inducing the weakest limb force (weak bedding system), were identified using MMT. Each bedding system, in total five mattresses and eight pillows of different firmness, was used for two continuous weeks at the participant's home in a random and double-blind sequence. A sleep log, a questionnaire, and a polysomnography were used to differentiate the two bedding systems. *Results and Conclusion*. Heart rate variability and arterial pressure variability analyses showed that the strong bedding system resulted in decreased cardiovascular sympathetic modulation, increased cardiac vagal activity, and increased baroreceptor reflex sensitivity during sleep as compared to the weak bedding system. Different bedding systems have distinct cardiovascular effects during sleep that can be predicted by MMT.

## 1. Introduction

Mattresses, as the sleep platforms, consist of their various types that have different physical properties, and therefore play an important role in rest efficiency. A proper bedding system is beneficial with respect to bodily health care, including both the cardiovascular system and an individual's sleep patterns. The effect of bedding systems on sleep quality has been demonstrated by both questionnaire and polysomnography [1, 2]. In terms of the cardiovascular system, the effects of bedding systems have been difficult to explore, and there are very few quantitative analyses available. However, because most people spend one-third of their life sleeping on a bed, if the bedding system has an effect on the cardiovascular system, the effect will be long-term and should not be neglected. Recent advances in the research methodology [3, 4], in relation to bedding systems, have provided an opportunity to research their effect on the cardiovascular neural regulation.

It is well known that sleep has dramatic effects on autonomic and cardiovascular functions [5]. For example, nonrapid eye movement (NREM) sleep is accompanied by an augmentation of vagal regulation, whereas rapid-eye-movement (REM) sleep is accompanied by activation of sympathetic functions [6–8]. Abnormal autonomic regulation during sleep has been linked to a disease model of spontaneous hypertension [9, 10]. Meanwhile, the relationship between sleep and health care of the cardiovascular system has also gained more attention in recent years. In addition, sleep-related factors such as respiration and even posture have an effect on autonomic functions [11]. Most young men (mean age 21.6 years in this study) have a preferred sleep position that is the actual position most often assumed during sleep [12]. In this context, lateral decubitus results in a lower sympathetic activity than supine decubitus [13].

The autonomic nervous system (ANS), which can be analyzed using electrocardiogram (ECG) monitoring, controls many aspects of the body's functions. Many researchers have begun to take into account the effect of ANS changes on various physiological situations as well as exploring the cause and effect relationships in some pathological conditions. When humans are under various types of stress, ANS disturbances are frequently induced and these may consist of suppressed vagal and/or enhanced sympathetic functioning [14–17]. In addition, baroreflex sensitivity, which can be analyzed using ECG and blood pressure signals, is able to be used to evaluate cardiovascular system central control [18, 19]. In freely moving humans or animals, the application of heart rate variability (HRV) and arterial pressure variability (APV) has recently gained popularity as a mean of quantifying autonomic functions noninvasively. HRV and APV have been categorized into high-frequency (HF) and low-frequency (LF) power according to their frequency [20–22]. HF of HRV is considered to represent vagal control of heart rate [20, 23]. The normalized LF (LF%) of HRV and LF of APV (BLF) are considered to reflect sympathetic modulations of the heart rate [20, 21] and blood vessels [9, 22, 24], respectively. ANS dysfunction and baroreflex impairment are known to be related to cardiovascular disorders [25–27], such as hypertension and acute coronary occlusion. Appropriate restoration of impaired ANS and baroreceptor reflex sensitivity (BRS) functioning, for example, by enhancement of vagal output, via an increased the BRS index, and by suppression of sympathetic functions, may be of benefit to the body.

A semiquantitative method using manual muscle testing (MMT) has been proposed as a classification method for bedding systems, including both the mattress and the pillow. The basis that underlies MMT is that the interactions between the body and the bedding system influence spinal function probably via compression of the circulation system of the spinal cord. Such spinal function can be revealed by its influence on the muscle power of the arms and legs. It has been demonstrated that a careful adjustment of an individual's bedding system using muscle power as a guide may be able to significantly relieve the severity of sleep-related respiratory problems [1]. We hypothesize that bedding selected by MMT may also affect the cardiovascular functions, Bedding systems using muscle power as a guide, should also have an effect on autonomic nervous functioning. Based on the above hypotheses, the present study was designed to provide a quantitative analysis of the effect of bedding systems on the cardiovascular neural regulation.

## 2. Materials and Methods

### 2.1. Subjects

The subjects participating in this study were ten healthy men with a mean age of 20.5 ± 0.3 years, an age range of 19–22 years, a mean body weight of 65.4 ± 1.7 kg, and a mean body mass index (BMI) of 21.9 ± 0.6 kg/m^2^. They were volunteer recruits from a university student population. All were in good health and had regular sleep/wake patterns (night sleep). There was no evidence of hypnotic drug abuse or above-average alcohol, caffeine, or nicotine consumption. None had a history of psychopathology or any medical condition known to influence sleep or the ANS. All subjects gave written informed consent after the experimental procedures were described to them. The procedures used in this study were approved by the Institutional Review Board of National Yang-Ming University. 

### 2.2. Bedding System and Muscle Testing

Similar to Tsai's study [1], five mattresses with various degrees of firmness (M1 to M5, the higher number meaning a higher grade of firmness, the materials including PTMS 5610, PTMS 5620, Sleep Tec Corporation, Taiwan), eight pillows of various heights (7 cm-to-9 cm), and two types of arc design (A and B) were provided by the manufacturer (Sleep Tec Corporation, Taiwan) and used in the present study. All of the mattresses were the same size (188 × 75 × 8 cm^3^).

MMT was performed by two senior staff members (2 years of muscle-testing experience) from the manufacturer. The muscles chosen for testing in the present study were the rectus femoris for mattress selection and the middle deltoid for pillow selection. To allow evaluation of each mattress surface, the participants were asked to lie on each surface and relax for a few seconds. Muscle tests on both sides of the rectus femoris were performed on all of the participants while they lay on each of the five mattress. Muscle tests were performed on the right middle deltoid with the participant lying on the mattress and initially using a pillow that had the least height. Each muscle test lasted less than 5 s, and three tests were performed for each muscle on each mattress or pillow with a 10–15 s rest interval between each test. The tester and participant were able to identify the selection of mattress and pillow that induced the strongest/weakest muscle forces at a rate >85%. The protocol for muscle test was described in detail in Tsai's study [1]. The ranks used for muscle strength to identify the strong and weak mattresses and pillows for each participant are presented in Table 1. In this study, 5/10, 4/10, and 1/10 of the participants had M2, M3, and M4 selected as the strong bedding system, respectively, and 4/10, 2/10, 3/10, and 1/10 of the participant had M4, M3, M2, and M1 selected as the weak bedding system, respectively (Table 1). A Chi-square test was used to compare the selection of categorical bedding systems and it was found that M2 and M3 were significant as strong bedding systems and M4 was significant as a weak bedding system. The selection of pillows was significant for 8 cm as the strong pillow and 7 cm and 9 cm as the weak pillows. 

### 2.3. Study Design

**Figure 1:**
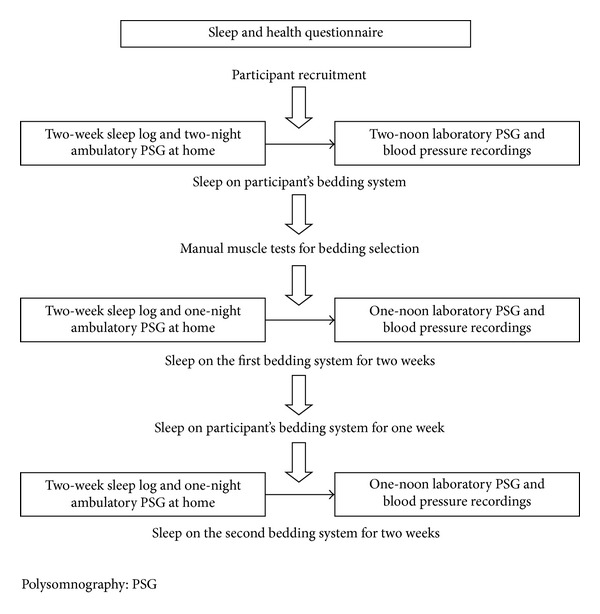
Study diagram.

Participants were initially evaluated using a two-week sleep log, the Pittsburgh sleep quality index (PSQI) and two nights of ambulatory polysomnography at home using the participants' own bedding system (mattress made out of coconut shell textile fiber or without a mattress). Next they were evaluated for 2 weeks using one of the two selected bedding systems and then for another 2 weeks using the other selected bedding system. There was a one week washout interval between the two bedding systems (Figure 1). Both the participants and the experimenters were blinded to the status of the bedding systems. During the 2 weeks of each trial at home of the bedding systems, information based on a two-week sleep log, the PSQI and one night of ambulatory polysomnography recording were collected. After the 2 weeks of each trial at home, the participants slept on the same bedding system in sleep laboratory for polysomnography (Embla, Inc., Broomfield, CO, USA) and arterial blood pressure (Finometer Model-1/Pro, FMS01, FMS, Inc., Amsterdam, Netherlands) recording at noon (more than one hour). During the washout period, the participants slept with their own pillows and mattress. Throughout the trial period, participants were asked to maintain their regular sleep schedules. 

### 2.4. Polysomnography

The signals used included central and occipital electroencephalograms (EEG), electrooculogram (EOG), chin and anterior tibialis electromyogram (EMG), ECG, blood pressure, and body position, which were recorded for more than one hour in the sleep laboratory. They were performed using a polysomnographic recorder (Embla, Inc., Broomfield, CO, USA) and an arterial blood pressure recorder (Finometer Model-1/Pro, FMS01, FMS, Inc., Amsterdam, Netherlands). Sleep stages, including active waking (AW), REM sleep, and NREM sleep were scored according to standard criteria that have been described elsewhere [28].

Ambulatory electrophysiological signals were recorded from each subject using a miniature physiological signal recorder (TD1, Taiwan Telemedicine Device Company, Taiwan). The small size (5.2∗3.1∗1.2 cm) and light-weight (11 gram) of the recorder produced minimal interference and stress [3]. The recordings started from 10 pm and lasted until waking. During the recording period, the subject was allowed to undertake normal daily activity except for vigorous physical exercise and they slept at their own homes. The recorded electrophysiological signals were a simplified version of standard sleep monitoring [28] with only four electrophysiological signals (EEG, EOG, EMG, and ECG) recorded. The EEG was recorded from the C3 point with a reference point at A2 [28]. The EOG was recorded from a pair of differential electrodes placed 1 cm above the right outer canthus and 1 cm below the left outer canthus [28]. This EOG recording is able to detect both horizontal and vertical movements of the eyeball in a single recording channel and is widely used in sleep research. Since the EOG was high pass filtered, it is sensitive to changes in the cornea position. The EMG was recorded from a pair of differential electrodes in the submental area. The ECG was recorded from the V2 site on the chest. The recorder was fixed to the chest wall by tape. The EEG, EOG, EMG, and ECG were amplified 2,000, 1,000, 1,000, and 250 fold, respectively [3]. The EEG was filtered between 0.34 Hz to 53 Hz, the EOG between 0.034 Hz to 53 Hz, the EMG between 16 Hz to 113 Hz and the ECG between 1.6 Hz to 113 Hz [3]. Finally the EEG, EOG, EMG, and ECG were synchronously digitized at a resolution of 12 bits but with different sampling rates (125, 125, 250, and 500 Hz, resp.) [3]. The acquired dataset was stored on a flash memory for subsequent off-line analysis.

### 2.5. Signal Processing and Data Analysis

For the sleep stage analysis, the data file was converted into European Data Format [29] and was then imported into a commercial sleep analysis software program (Somnologica 3.1.2, Embla, USA). Computer assisted sleep analysis was carried out according to the criteria defined by Rechtschaffen and Kales and the American Academy of Sleep Medicine [28]. The results were verified by a qualified sleep technician. All polysomnographic data were manually scored in 30 s epochs. Sleep variables such as total sleep time, time and percent of total sleep time taken up by REM sleep, NREM sleep and their substages, sleep latency, sleep efficiency (percent of total sleep time), and number of stage changes were calculated.

For the analysis of the EEG and ECG data, we designed a special computer program in Pascal language (Borland Pascal 7.0, Borland, USA). The details of the computer program are as follows. Preprocessing of the ECG signals was designed according to the recommended procedures [20] as detailed in our previous investigations [6, 30]. In brief, the computer algorithm identified each QRS complex and rejected each ventricular premature complex or noise according to its likelihood using a standard QRS template. Stationary R-R intervals (RR) were resampled and linearly interpolated at a rate of 64 Hz to provide continuity in the time domain. The sampling rate of the EEG signal was also reduced to 64 Hz by averaging. The amplitudes of the EEG, APV, and HRV were measured in the frequency domain using power spectral analysis. The EEG, arterial pressure, and RR signals to be analyzed were truncated into successive 64-s (4,096 points) time segments (windows or epochs) with 50% overlap. A Hamming window was applied to each time segment to attenuate the leakage effect [31]. Our algorithm then estimated the power density of the spectral components based on fast Fourier transformation. The resulting power spectrum was corrected for attenuation resulting from sampling and the application of the Hamming window [30].

The detailed analytical procedures for APV and HRV have also been described previously [9, 10, 24, 31, 32]. Briefly, mean AP (MAP) was obtained by the integration of the digitized AP signals. The mean of RR were estimated continuously from the digitized ECG signals. Stationary MAP and RR were resampled and interpolated to provide continuity in the time domain. These sequences were analyzed using fast Fourier transformation after the application of a Hamming window [31]. For each time segment, BLF (0.04–0.15 Hz) of the AP spectrogram, HF (0.15–0.4 Hz), and LF% (0.04–0.15 Hz) of the RR spectrogram were quantified. BLF, LF%, and HF provided markers of sympathetic vasomotor control, cardiac sympathetic modulation, and cardiac vagal activity, respectively [20–22, 32].

Spontaneous BRS was estimated from the AP-RR transfer function and AP-RR linear regression as described previously [9, 24, 32]. In brief, for the transfer function analysis, the transfer magnitude at high-frequency (BrrHF) and low-frequency (BrrLF) ranges were estimated. For the linear regression analysis, the slope of the linear regression between the AP and RR pairs that were ascending simultaneously was estimated as the BrrA. The slope of the linear regression between AP and RR pairs that were descending simultaneously was estimated as the BrrD. The APV, HRV, and BRS parameters of each sleep-wake state were averaged for each individual subject.

### 2.6. Sleep Log

Similar to the Tsai's study [1], there were two portions to the sleep log. The first portion was completed within 5 min after getting up from bed in the morning. This portion of the sleep log had nine visual analog scale (VAS) questions regarding sleepiness, alertness, degree of comfort with regard to the mattress, degree of comfort with regard to the pillow, neck or shoulder tenderness, back tenderness, degree of satisfaction with the bedding system, quality of sleep, and degree of satisfaction with sleep. The other portion was completed in the 10 min before sleeping at night. This portion of sleep log had five VAS questions regarding daytime alertness, degree of comfort with regard to the mattress, degree of comfort with regard to the pillow, and current sleepiness and alertness. Each VAS question consisted of a 10 cm line with extreme polar labels at each side of the line. Ticks and tick labels every 20 mm were present under the line of the last question. The participants then placed a single mark on each line according to their scores.

### 2.7. Statistical Analysis

For each time segment, the LF and HF of the RR spectrogram [20, 30] and the BLF were quantified by integration, that is, by calculation of the area of the power spectral density between two specified frequencies. The distributions of the HF and LF were not normal but were skewed to the right; therefore, the HF and LF were logarithmically transformed to correct for the distribution skewness [30]. Significant differences across groups were assessed using analysis of variance (ANOVA) for repeated measures. When indicated by a significant *F* statistic, regional differences were isolated using *post hoc* comparisons by the Fisher's least significant difference test. Significant differences between the values and zero were confirmed with a 95% confidence interval analysis. Comparisons between two sets of data were performed using the paired *t* test. Statistical significance was assumed for *P* < 0.05. Data are presented as means ±  SEM.

## 3. Results

### 3.1. The Effect of the Selected Bedding Systems on Sleep Architecture

In this study, in terms of sleep quality evaluation, we chose to use the subjective questionnaire and the objective polysomnographic recording to gather the sleep quality parameters for comparison. The subjects were recorded at their home for two nights and we used the data from the second night to avoid any first night effect. In addition, we recorded the same parameters on the same day of the week. For each participant individually, two bedding systems, one inducing stronger muscle forces and the other inducing weaker muscle forces, were selected. The subjective sleep quality data from the PSQI revealed that the sleep latency for the selected strong and weak bedding systems was significant lower than for their own bedding system (Table 2). The objective sleep quality data from the ambulatory polysomnography system recorded in their own home revealed that the number of arousals, arousal index, and the time of stage 2 during sleep were significantly different for the selected weak and strong bedding systems compared to their own bedding system (Table 2). 

### 3.2. The Effect of the Selected Bedding Systems on the Subjective Evaluation of Other Variables

The nine VAS questions, made up of sleepiness, alertness, degree of comfort with regard to the mattress, degree of comfort with regard to the pillow, neck or shoulder tenderness, back tenderness, degree of satisfaction for the bedding system, quality of sleep, and degree of satisfaction in sleep, were not significant different between the weak and strong bedding systems. 

### 3.3. The Effect of the Selected Bedding Systems on Cardiovascular Neuronal Regulation

**Figure 2:**
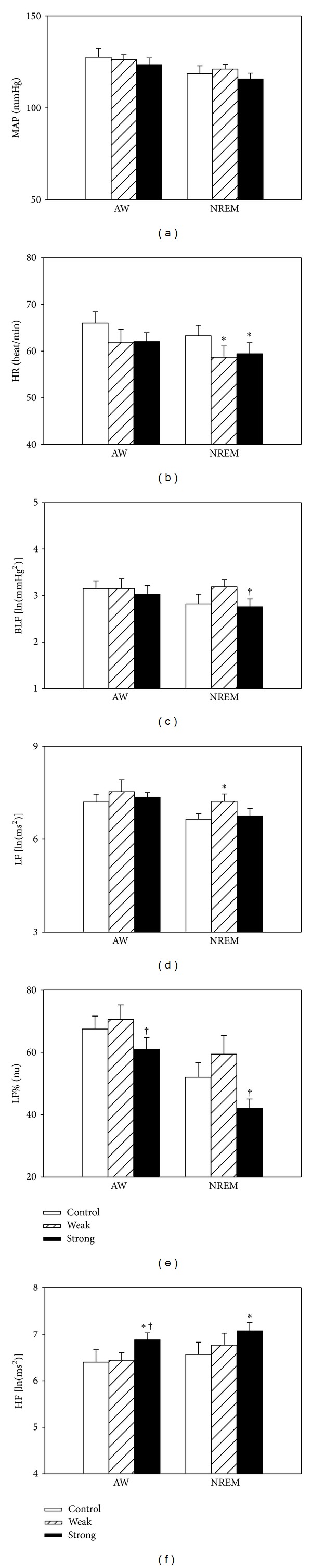
Comparisons of MAP, HR, BLF of APV, LF, HF, and LF% of HRV across the control, selected weak bedding system, and selected strong bedding system during AW and during NREM from noon laboratory PSG and blood pressure recordings. The values are presented as means ±  SEM; *n* = 10. *P < 0.05 versus the control bedding system. ^†^ *P* < 0.05 versus the selected weak bedding system by Fisher's least significant difference test. ln, natural logarithm; nu, normalized units.

**Figure 3:**
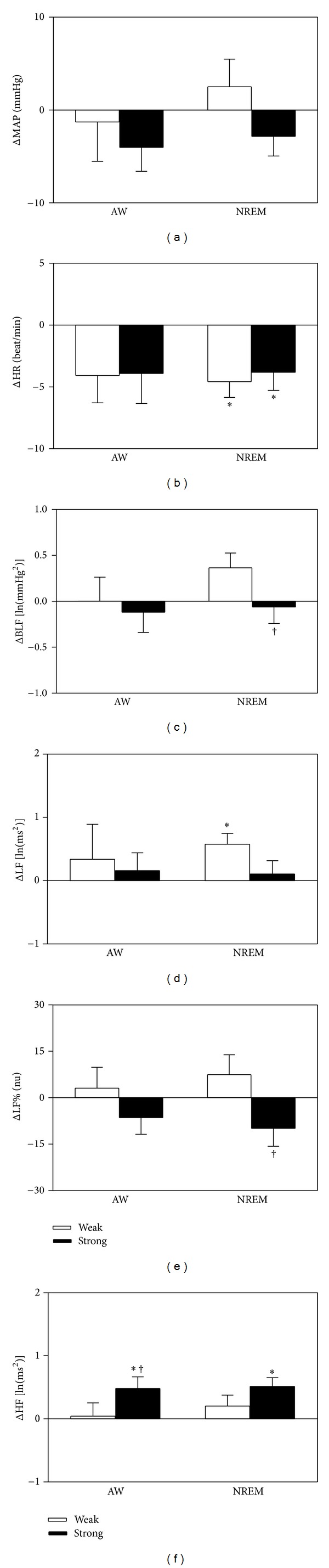
The changes (Δ) in MAP, HR, BLF of APV, LF, HF, and LF% of HRV across the selected weak and strong bedding systems relative to the control (participant's) bedding system during AW and NREM from noon laboratory PSG and blood pressure recordings. The values are presented as means ±  SEM; *n* = 10. *P < 0.05 versus zero by 95% confidence interval analysis. ^†^ *P* < 0.05 versus the selected weak bedding system by paired *t* test. ln, natural logarithm; nu, normalized units.

Due to the presence of significant sleep/wake related ANS changes, we divided the autonomic nervous activity of the individuals based on their sleep/wake status. We also used power spectral analysis and cross-spectral analysis of blood pressure and heart rate to analyze the cardiovascular ANS parameters without any further disturbance. When compared to the control bedding system, the cardiac vagal activity (HF), both during AW and during NREM, was significantly increased with the strong bedding system. Both bedding systems showed a lower heart rate during NREM. When compared to the weak bedding system, the cardiovascular sympathetic activity (LF% and BLF) during NREM and the HF during AW were significantly decreased and increased, respectively, for the strong bedding system (Figure 2). When compared to zero, the delta change of the heart rate, relative to the control bedding system during NREM, was significantly decreased for the strong and weak bedding systems. The delta change of the LF, relative to the control bedding system during NREM, was significantly increased with the weak bedding system and the delta changes of the HF, relative to the control bedding system during AW and NREM, were both significantly increased with the strong bedding system (Figure 3). However, when compared to the weak bedding system, the delta change of cardiovascular sympathetic activity (LF% and BLF), relative to the control bedding system during NREM, was significantly decreased. Furthermore, the cardiac vagal (HF) activity during waking was significantly increased with the strong bedding system (Figure 3). 

**Figure 4:**
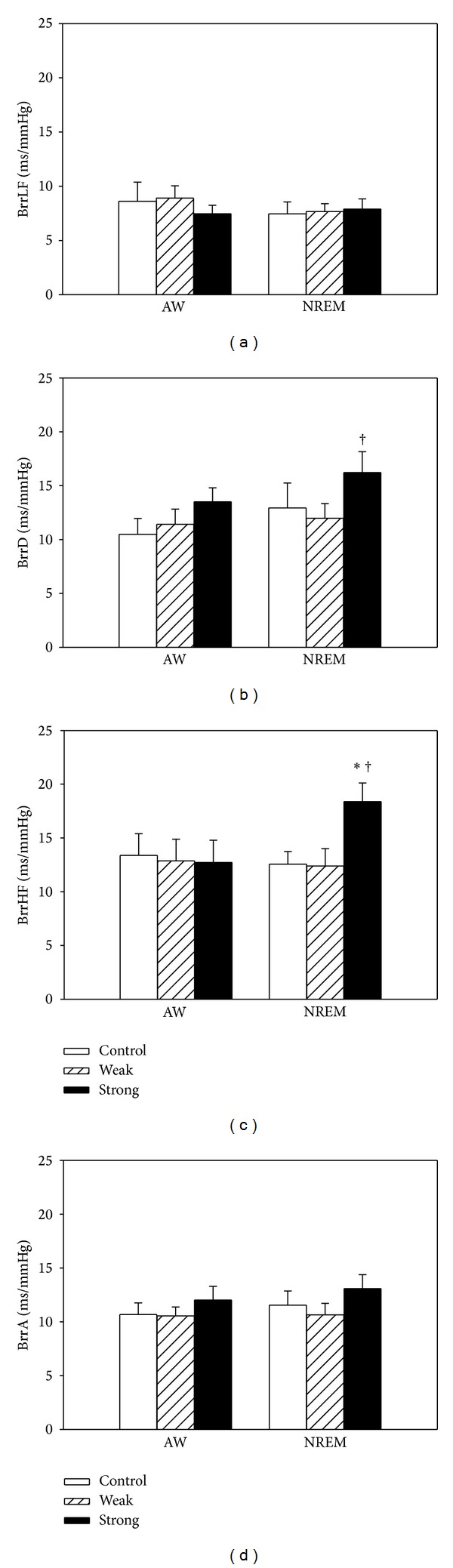
Comparisons of spontaneous baroreflex sensitivity across the control, selected weak, and selected strong bedding systems during AW and NREM from noon laboratory PSG and blood pressure recordings. Four indices were used for this analysis: magnitude of transfer function between MAP and RR signals over the frequency ranges of 0.04–0.15 Hz (BrrLF) and 0.15–0.4 Hz (BrrHF) together with the slope of the linear regression between MAP and RR pairs under successively descending (BrrD) and ascending (BrrA) changes. Values are presented as means ±  SEM; *n* = 10. *P < 0.05 versus the control bedding system. ^†^ *P* < 0.05 versus the selected weak bedding system by Fisher's least significant difference test.

**Figure 5:**
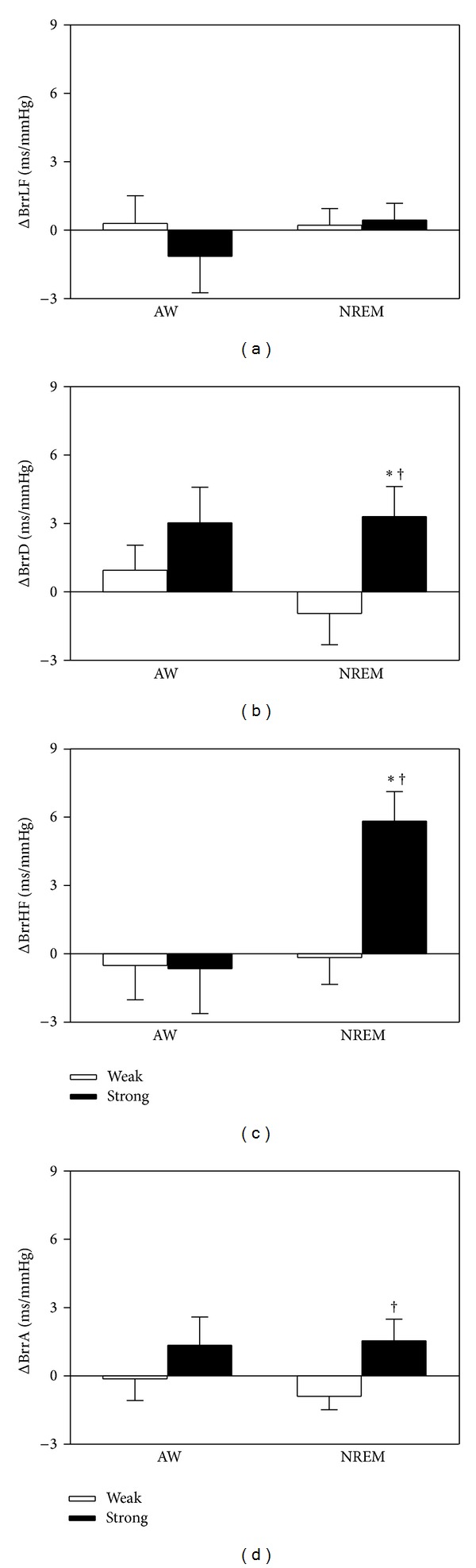
The changes (Δ) in the magnitude of the MAP-R-R intervals transfer functions (BrrHF, BrrLF) and the slopes of mean arterial pressure-R-R intervals linear regressions (BrrA, BrrD) with the selected weak and strong bedding systems relative to the control (participant's) bedding system during AW and NREM from noon laboratory PSG and blood pressure recordings. Values are presented as means ±  SEM; *n* = 10. *P < 0.05 versus zero by 95% confidence interval analysis. ^†^ *P* < 0.05 versus the selected weak bedding system by paired *t* test.

The baroreceptor reflex index (BrrHF) during NREM was significantly increased for the strong bedding system compared to either the control or weak bedding systems. The BrrD during NREM was significantly increased for the strong bedding system compared to the weak bedding system (Figure 4). When compared with zero, the delta changes of the BrrHF and BrrD with the control bedding system during NREM were significantly increased with the strong bedding system. However, as compared with the selected weak bedding system, the delta changes of the BrrHF, BrrD, and BrrA, relative to the control bedding system during NREM, were significantly increased with the strong bedding system (Figure 5).

## 4. Discussion

MMT is practiced clinically in order to assess neuromuscular functioning in neurology and related departments [33, 34]. The idea of applying MMT for bedding selection originates from Tsai and Liu [1]. They found that appropriate selection results in fewer sleep-related respiratory problems in subjects with mild sleep-related respiratory disturbances. In the current study, the bedding system can be divided into two bedding systems (strong bedding system and weak bedding system) according to the waking MMT test, which was a double blind procedure. The healthy young men then slept on each bedding system for two weeks and their subjective and objective sleep qualities were compared between the two systems. The sleep analyses were accomplished by the polysomnographic technique using EEG, EOG, and EMG signals. Changes in cardiovascular autonomic functions and baroreflex sensitivity were quantified noninvasively by frequency domain analyses of HRV and APV. We used power spectral analysis and cross-spectral analysis of blood pressure and heart rate to analyze cardiovascular functions without disturb the subjects during sleep. Compared to the weak bedding system, two weeks of sleeping on the strong bedding system significantly promoted baroreflex sensitivity, enhanced cardiovagal activity, and attenuated cardiovascular sympathetic functioning. 

These findings imply that a bedding system is able to affect human cardiovascular neural regulation and also showed that the application of MMT is effective at predicting such effects. These effects occurred under conditions where the mean blood pressure, mean heart rate, sleep quantity, and sleep quality were not significantly different. Since the selected weak and strong bedding systems were not associated with various subjective variables, including the degree of comfort, degree of satisfaction, contact temperature, and the degree of support to the body, these variables ought not to be acting as confounders relative to the effects. Furthermore, the test results for sleep-related cardiovascular functioning are significantly related whether the selected system is a “fit” or “unfit” bedding system.

This study is the first to show that two weeks of sleeping on a strong bedding system, as selected by MMT, seems to be able to significantly change cardiovascular neuronal regulation. The beneficial effects included a significant decrease in sleep related cardiovascular sympathetic activity, a significant increase in sleep related BRS indexes, and a significant increase in waking related cardiac vagal activity. These are novel and objective findings showing that the selection of a bedding system using MMT approach can directly affect cardiovascular functioning. Notwithstanding the present findings, it should be noted that the effects of bedding systems on older subjects or subjects suffering from disease still need to be explored.

Our testing system classified the subjects' sleep/wake states because there are drastic changes in ANS and BRS across the various sleep/wake states [9, 35]. In order to detect the autonomic functioning continuously as well as to avoid interfering with the subjects' normal physiological cycles and autonomic functions, the HRV and APV analyses were carried out noninvasively [6, 9]. During sleep, the functional connection between the central nervous system and the outside environment is attenuated, which results in a minimization of the influence of the external environment on the body. Therefore, investigation during sleep ought to provide a more specific measurement of functioning inside the body and the findings should be minimally affected by body-environment interaction [9, 36]. Most people spend one-third of their life sleeping on a bed, thus it is clear that more attention needs to be paid to the effects of the bedding system on the ANS.

A previous study demonstrated that, as compared to a bedding system inducing a weaker muscle power, a bedding system inducing a stronger limb muscle power is able to decrease the incidence of sleep-related respiratory disturbance [1]. One possibility is that the strong bedding system maintains the head in a more appropriate posture and/or moves the spine into a better alignment, particularly when subjects suffer from sleep-related respiratory disturbances due to mechanical causes [1]. Sleep-related respiratory disturbances have been shown to evoke sympathetic excitation, which may result in hypertension [37–39]. In this study, we excluded two subjects who were found to have unpredicted sleep-related respiratory disturbances during the experiment. The present study demonstrated that two weeks sleep in a strong bedding system is able to decrease cardiac sympathetic activity. Although the subjects were healthy men, it is likely that our results indicate a logical connection between the strong bedding system and low sympathetic activity, which, in turn, could be involved in improving sleep-related respiratory disturbance. On the other hand, previous studies have suggested that sleep position has a significant effect on cardiac ANS [13, 40]. Kubota et al. [12] determined that most of young men (mean age 21.6 years) have a preferred sleep position that is the actual position these individuals assumed during sleep most often. Therefore, in the present study, we have assumed that the sleep position of our individual subjects does not vary from night to night. Although the sleep position during polysomnographic recording in this study was not changed, the selected bedding system may have changed the subjects' habitual sleep position and this might have affected their ANS. However, notwithstanding whether the sleep position of individual subjects has changed or not, the final outcome of the strong bedding system on our subjects was a significant improvement in cardiovascular functioning.

The firmness of the strong bedding system in this study is different from that of the weak bedding system (Table 1). It is possible that the change in the firmness of the mattresses may directly affect the compression pressure affecting over the lung, heart and vessels [41]. In addition, the compression of these thoracic organs has been reported to influence cardiac vagal activity [13]. Since the selection of the weak and strong bedding systems was double blinded and was not associated with a subjective evaluation using other variables, including the degree of comfort, degree of satisfaction, contact temperature, and the degree of support to the body, these subjective feelings ought not to be confounders with respect to the results of this study. However, there remains the possibility that ANS and sleep may interact based on animal evidence [8, 42, 43], even though the present study shows that sleep quality and quantity do not significantly change between the weak and strong bedding systems.

A more detailed examination of bed preference and the sustained effect of different bedding systems need to be carried out. Furthermore, whether different gender and age, in relation to the bedding system, have an effect on cardiovascular regulation and sleep patterns also warrants further exploration. Since all subjects needed to participate in the experiment for at least one month and we excluded individuals with sleep breathing and cardiovascular disorders that may affect ANS activity, it was different to obtain a large number of suitable volunteers for the present study. Thus another limitation of this study is that the effective sample size is relatively small. An increased number of subjects is needed in any future studies. Finally, we did not test MMT using control bedding system and therefore cannot make any comparison in terms of muscle power between the control and MMT selected bedding systems.

The present study reveals that, between the weak and strong bedding systems selected by MMT, while there are significant differences in cardiovascular neuronal regulation, there are no significant changes in sleep quantity and quality. As compared to the original bedding system, the participants, when they used the two new bedding systems, one of which was weak and the other being strong, there was a shortened sleep latency. The new bedding systems further demonstrated decreased sleep arousal. One possibility is that most of the control bedding systems used by the students are poorer in quality than either of the new bedding systems. As a result, sleep quality may have increased when they slept on both of the new systems. Another possible reason is the psychological effect of having a new bedding system. Our results are unable to explain the reductions in sleep stage 2, arousal and sleep latency of PSQI after sleeping on the new bedding systems. Finally, the applied polysomnographic recorder was small and easily used for ambulatory recording; furthermore, the recordings were performed for all the three bedding systems. Therefore, the sleep recording itself should not be a confounding factor.

## 5. Conclusion

Our results suggested that MMT is a useful approach to select a bedding system that can affect cardiovascular regulation. Bedding system selection based on MMT has an impact in ANS activity during sleep. Sympathetic tone was decreased and cardiovagal tone was increased during NREM sleep as measured by HRV and APV. The strong bedding system based on MMT is able to improve cardiovascular modulation during sleep in healthy young men.

## Figures and Tables

**Table 1:** Muscle strength ranks for the strong/weak mattresses and pillows for each participant. All five mattresses and eight pillows were tested and ranked by the tester.

Participant no.	Mattresses		Pillows	
	Weak	Strong	Weak	Strong
	Code	Code	Code	Code
1	M4	M3	9B	8A
2	M3	M2	7B	8A
3	M1	M4	7B	8A
4	M3	M2	7A	8B
5	M4	M2	9A	8B
6	M2	M3	9A	8B
7	M2	M3	9B	8A
8	M4	M2	9A	8B
9	M2	M3	9B	8A
10	M4	M2	9A	8B

**Table 2:** Summary of the polysomnographic data and PSQI scores collected at each stage during the 2-week trials of the control, weak and strong bedding systems.

Parameters	Control	Weak	Strong
Total sleep time (min)	440.6 ± 20.7	379.3 ± 19.2	396.6 ± 23.1
Sleep latency (min)	9.7 ± 3.1	10.9 ± 4.3	16.8 ± 4.1
NREM stage 1 sleep (min)	24.8 ± 3.0	24.3 ± 2.4	20.2 ± 2.9
NREM stage 2 sleep (min)	237.3 ± 17.1	196.4 ± 13.2*	202.3 ± 16.4*
Slow wave sleep (min)	51.2 ± 7.6	57.1 ± 11.6	53.5 ± 5.8
REM sleep (min)	127.0 ± 9.8	101.6 ± 8.6	120.8 ± 9.7
Wake after sleep onset (min)	30.2 ± 3.3	31.3 ± 5.5	27.4 ± 4.3
Sleep efficiency (%)	91.7 ± 0.9	90.2 ± 1.5	89.9 ± 1.2
NREM stage 1 sleep (%)	5.6 ± 0.6	6.6 ± 0.8	5.1 ± 0.7
NREM stage 2 sleep (%)	53.5 ± 1.9	52.0 ± 2.7	50.6 ± 1.7
Slow wave sleep (%)	12.2 ± 2.0	14.7 ± 2.7	13.9 ± 1.6
REM sleep (%)	28.7 ± 1.5	26.7 ± 1.9	30.5 ± 1.7
Number of arousals in sleep	45.2 ± 7.1	30.6 ± 2.7*	28.7 ± 3.0*
Arousal index in sleep	6.2 ± 1.0	4.8 ± 0.4*	4.3 ± 0.3*
Global PSQI	5.7 ± 0.8	4.0 ± 0.5	3.6 ± 0.4
Sleep latency of PSQI	1.3 ± 0.2	0.6 ± 0.2*	0.8 ± 0.1*
Sleep disturbance of PSQI	0.9 ± 0.2	1.1 ± 0.3	0.4 ± 0.2

Data for the weak and strong bedding systems were from ambulatory polysomnographic recordings. Values are presented as means ± SEM; *n* = 10. *P < 0.05 versus the participant's control bedding system.
